# Integrating noise as a risk factor in studies of Alzheimer's disease and dementia: Guidance for epidemiologic research

**DOI:** 10.1002/alz.71513

**Published:** 2026-05-22

**Authors:** Matthew Bozigar, Jennifer Weuve, Sierra N. Clark, Stephanie T. Grady, Manuella Lech Cantuaria, Yutong Samuel Cai, Charlotte Roscoe, Martin Röösli, Junenette L. Peters, Edmund Seto, Danielle Vienneau, Mette Sørensen, Richard L. Neitzel, Aaron L. Hastings, Thomas J. Luben, Zorana Jovanovic Andersen, Charlotte Clark, Anna L. Hansell, Perry Hystad, Michael Brauer, Sara D. Adar

**Affiliations:** ^1^ School of Nutrition and Public Health, College of Health Oregon State University Corvallis Oregon USA; ^2^ Department of Epidemiology Boston University School of Public Health Boston Massachusetts USA; ^3^ Department of Population Health & Policy, School of Health and Medical Sciences City St George's, University of London London UK; ^4^ Department of Environmental Health Boston University School of Public Health Boston Massachusetts USA; ^5^ Work, Environment and Cancer Danish Cancer Institute Copenhagen Denmark; ^6^ Research Unit for ORL—Head & Neck Surgery and Audiology University of Southern Denmark Odense Denmark; ^7^ Department of Collective Health, Faculty of Medical Sciences State University of Campinas Campinas Brazil; ^8^ Centre for Environmental Health and Sustainability University of Leicester Leicester UK; ^9^ NIHR Health Protection Research Unit in Chemical Threats and Hazards University of Leicester Leicester UK; ^10^ NIHR Leicester Biomedical Research Centre (BRC) Leicester UK; ^11^ Environmental Systems and Human Health Oregon Health & Science University‐Portland State University (OHSU‐PSU) School of Public Health Portland Oregon USA; ^12^ Swiss Tropical and Public Health Institute Allschwil Switzerland; ^13^ University of Basel Basel Switzerland; ^14^ Department of Environmental and Occupational Health Sciences, School of Public Health University of Washington Seattle Washington USA; ^15^ Julius Center for Health Sciences and Primary Care University Medical Center Utrecht Utrecht the Netherlands; ^16^ Department of Natural Science and Environment Roskilde University Roskilde Denmark; ^17^ School of Public Health University of Michigan Ann Arbor Michigan USA; ^18^ Volpe National Transportation Systems Center U.S. Department of Transportation Cambridge Massachusetts USA; ^19^ Department of Public Health University of Copenhagen Copenhagen Denmark; ^20^ Leicester British Heart Foundation Centre of Research Excellence Leicester UK; ^21^ School of Population and Public Health The University of British Columbia Vancouver British Columbia Canada; ^22^ Institute for Health Metrics and Evaluation University of Washington Seattle Washington USA

**Keywords:** cognition disorders, environment design, environmental exposure, public health, risk assessment, spatial analysis

## Abstract

Noise exposure is increasingly recognized as a modifiable environmental risk factor for Alzheimer's disease and related dementias (ADRD), yet its integration into epidemiologic research remains limited. We reviewed international noise mapping resources, exposure metrics, and analytic approaches relevant to ADRD studies. Mechanistic pathways and methodological challenges were synthesized from recent studies via expert knowledge. We present a stepwise framework for integrating noise into ADRD research, detailing metric selection, spatiotemporal assignment, and analytic guardrails. Our review recommends the application of 24‐hour average, nighttime, and event‐based metrics, and stresses that health effects may occur at low noise levels. We further underscore the importance of accounting for indoor, occupational, and life course exposures. Rigorous noise exposure assessment and transparent reporting will improve comparability and causal inference in ADRD studies. Future research should harmonize exposure metrics, integrate co‐exposures (e.g., air pollution), and clarify etiologically relevant windows to strengthen prevention strategies.

## HIGHLIGHTS

- This review identifies research gaps in exposure–response at low noise levels.
- A stepwise framework for noise exposure in ADRD research is presented.
- We emphasize metric selection and assignment to minimize misclassification.
- Global noise mapping resources and analytic guardrails are reviewed.
- Integrating indoor, occupational, and life course exposures is recommended.

## 1 INTRODUCTION

Noise is an overlooked but modifiable risk factor with several plausible mechanistic links to Alzheimer's disease and related dementias (ADRD).^1^, ^2^, ^3^ Emerging evidence from large cohort studies has demonstrated links between chronic environmental noise exposure and incident ADRD and mild cognitive impairment (MCI).^1^, ^2^, ^4^, ^5^ Hundreds of millions of people are exposed to high levels of transportation noise in the United States and Europe alone.^6^, ^7^ Yet historically, noise has been relatively understudied compared to air pollution, despite the high exposure prevalence globally, and the potential for both independent and synergistic health effects.^2^, ^5^

Some soundscapes, which encompass all sounds in an environment, can be conducive to healthy aging. Sounds such as birdsong, flowing water, or music may have calming effects or cognitive benefits.^8^, ^9^, ^10^ Indeed, the health impact is likely context dependent—for example, music or natural sounds may be pleasing in one setting and time but intrusive in others.^8^, ^9^ Noise is defined as sound that is either unwanted or harmful.^11^, ^12^ All noise occurring in a person's surroundings is termed environmental noise, whereas community noise is the totality of noise occurring outside in a person's residential community. Transportation noise, a major component of community and environmental noise, refers to outdoor sounds from sources such as road traffic, aircraft, and railways. It is the dominant outdoor noise source in most high‐income countries, with road traffic most prevalent, while aircraft and railway noise tend to be more intermittent and disruptive.^13^, ^14^ Other sources of noise include occupational and industrial settings, where some of the highest noise exposures occur, as well as indoor, neighbor, and recreational sources.^15^, ^16^, ^17^

Environmental noise exposure is widespread in the general population globally. It is estimated that > 100 million Europeans are exposed to high levels of road traffic noise.^13^ More broadly, according to the 2025 European Union (EU) Environmental Noise Directive (END), > 20% of Europeans (110 million) are exposed to transportation noise levels > 55 A‐weighted decibels (dBA) for day–evening–night average sound level (L_den_).^18^ When contrasted with the World Health Organization (WHO) recommendations (53 dBA for road traffic, 54 dBA for railway, and 45 dBA for aircraft noise using L_den_),^13^ this figure rises to > 30%, or nearly 150 million Europeans. In the United States, the Bureau of Transportation Statistics estimates that ≈ 74 million Americans (≈ 23% of the population) were potentially exposed to noise ≥ 50 dBA from aviation and road sources in 2018,^19^ and 100 million experience ≥ 70 dBA of noise from all sources combined.^20^ In New York City, 90% of residents are exposed to ≥ 70 dBA of all‐source noise.^21^ Recent studies conducted in African and Asian cities also reported average environmental noise levels well above WHO guidelines. For example, almost all residents in Accra, Ghana, live in areas with environmental noise above WHO guidelines for road‐traffic noise^22^ and close to 70% of the population in Kigali (Rwanda) live in areas above national standards.^23^ In Taichung, Taiwan, annual mean 24‐hour and nighttime levels were 66.4 and 62.1 dB, respectively;^24^ researchers in Shanghai recorded an annual mean of 62 dB,^25^ and Guangzhou averaged 65.8 dB, with daytime levels ≥ 67 dB.^26^ A review of Arab countries highlighted widespread urban noise pollution but limited systematic exposure data.^27^ It has also been demonstrated that, like many environmental stressors, noise exposure presents environmental justice challenges, in which higher noise exposure burdens fall disproportionately on disadvantaged and marginalized communities.^5^, ^28^, ^29^

**FIGURE 1:**
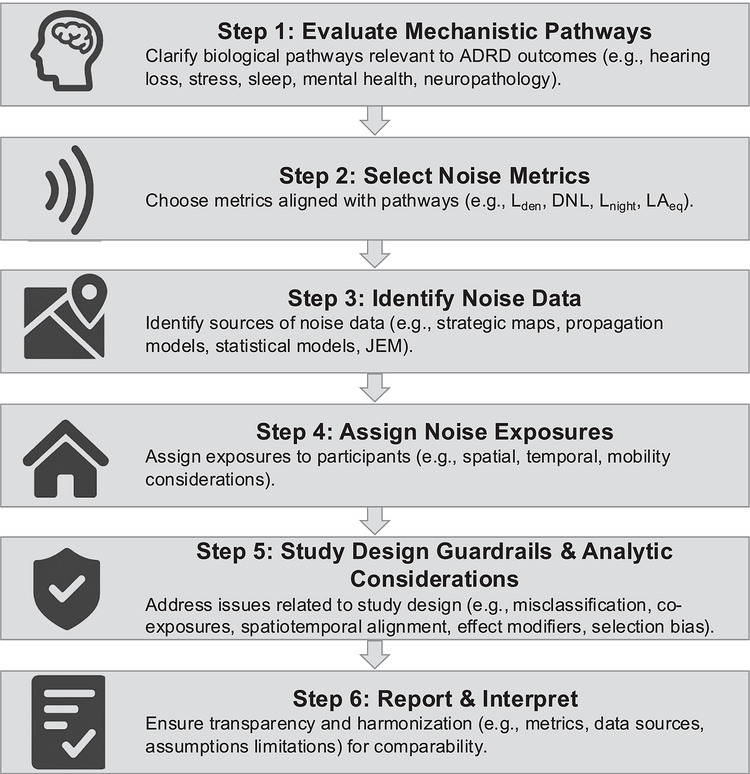
Proposed stepwise approach for incorporating noise as a risk factor in Alzheimer's disease and related dementias (ADRD) research

To support ADRD researchers in incorporating noise as an exposure in research, we provide guidance rooted in current evidence and best practices. Our focus is on general‐population and cohort‐based epidemiologic studies, in which noise is typically assigned based upon residential locations using geospatial models or existing noise maps rather than direct personal measurement. This step‐by‐step guide (Figure 1) begins by first helping identify key mechanistic pathways appropriate for an ADRD outcome based on previous epidemiologic evidence in Step 1. Step 2 follows, in which we suggest noise metrics by mechanistic pathway. In Step 3, we outline practical strategies for identifying and obtaining noise data, and in Step 4, we detail considerations when assigning noise exposures to study participants. We next provide guidance on study design and analytic considerations in Step 5, along with reporting considerations to enhance rigor, comparability, and interpretability in ADRD noise research. We end with Step 6, summarizing the reporting and interpretation of study results.

This work was led by the Gateway Exposome Coordinating Center (GECC), an interdisciplinary research consortium established to advance research on environmental exposures across the life course (i.e., exposome) that influence ADRD.^30^ The GECC aims to foster collaboration and consensus building and to develop and share exposome data, tools, and measures to support ADRD research. Guided by these objectives, we established an expert working group, engaged a wide array of noise researchers, and synthesized knowledge and recommendations through collaborative discussion and consensus building.

## 2 STEP 1: EVALUATING MECHANISTIC PATHWAYS OF INTEREST IN THE CONTEXT OF EPIDEMIOLOGICAL EVIDENCE

**FIGURE 2:**
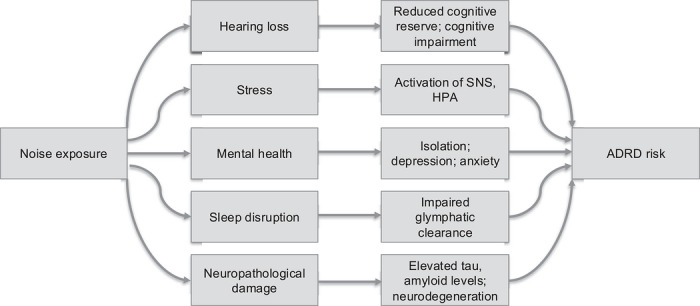
Key plausible mechanistic pathways for noise exposure and Alzheimer's Disease and Related Dementia outcomes. Included pathways are not exhaustive of all possible pathways. Interactions are not included for simplicity, though we note that many pathways are interrelated and may indeed exhibit complex interaction patterns. ADRD, Alzheimer's disease and related dementias; HPA, hypothalamic–pituitary–adrenal axis; SNS, sympathetic nervous system.

There are several plausible and demonstrated mechanistic pathways supported by epidemiological evidence through which noise exposure may affect ADRD (Figure 2). We focus here on five main pathways, recognizing that these are not exhaustive and are interrelated. The pathways discussed here are: (1) hearing loss, (2) stress, (3) mental health, (4) sleep, and (5) neuropathological damage. Researchers should concisely state their research question to identify the putative pathway(s) through which noise might affect ADRD, as this choice guides decisions about noise metrics, data needs, study design, and analysis.

At high levels, which are often present in occupational settings and can be produced by transit use and transportation sources in dense urban areas, chronic noise exposure can cause auditory or hearing damage and hearing loss, which can limit social connectivity and reduce intellectual stimulation, both of which may contribute to cognitive decline.^21^, ^31^, ^32^ Midlife hearing loss has been suggested as one of the leading modifiable risk factors for ADRD.^33^ Chronic noise exposure can also contribute to stress and activate the sympathetic nervous system (SNS) and hypothalamic–pituitary–adrenal (HPA) axis, which elevates cortisol concentrations.^34^, ^35^ Chronically elevated cortisol is further linked to increased risk of hypertension, stroke, and other cardiovascular diseases^36^, ^37^, ^38^, ^39^ that are risk factors for ADRD. Chronic annoyance due to noise, especially due to sleep disruption, as well as noise‐induced stress, has been associated with anxiety, depression, and social isolation.^40^, ^41^ These aspects of mental health are independently linked with ADRD incidence.^42^, ^43^ Yet, there is also evidence of soundscapes protective of mental health. Nature sounds such as birdsong and flowing water, as well as music therapy, have been associated with reduced agitation and improved mood and well‐being in people with ADRD.^8^, ^44^ Another important mechanism by which noise may impact ADRD is sleep disturbance, usually driven by nighttime noise exposure. Slow‐wave sleep is particularly disrupted by noise, which can impair glymphatic clearance, an important process in daily brain recovery.^45^, ^46^ Importantly, noise can disturb sleep even at modest levels.^47^, ^48^ However, pink noise (having equal energy per octave rather than white noise having equal energy per frequency) may enhance slow‐wave sleep and memory consolidation,^49^ and frequency‐specific brain stimulation has been shown to promote glymphatic clearance in mouse models.^50^ Finally, chronic noise exposure can also induce physiological changes to the brain through processes related to oxidative stress, neuroinflammation, and disruption of the blood–brain barrier.^51^, ^52^ In fact, there is evidence that animals exposed to chronic noise develop neurofibrillary tangles and hyperphosphorylation of tau in key areas of the brain needed for learning and memory.^53^ Chronic noise exposure has also been associated with hippocampal atrophy in animal models.^54^ While the human epidemiologic evidence linking noise to ADRD is growing, it remains largely observational. In contrast, a body of in vivo animal studies provides more direct evidence for a causal contribution of chronic noise exposure to cognitive impairment and AD‐like neuropathology, including tau hyperphosphorylation,^53^ amyloid beta accumulation and neuroinflammation,^55^ and accelerated pathology in transgenic AD models^56^ (see Table S1 in supporting information for a comprehensive summary). Such experimental findings strengthen biological plausibility and support the mechanistic pathways outlined above.

The epidemiologic literature on noise and ADRD is still sparse and somewhat mixed, but recent studies have begun to generate compelling evidence of the risk posed by noise exposure for adverse ADRD outcomes. For example, large, prospective cohort studies in Denmark and the UK demonstrated elevated risk of incident dementia from transportation noise exposure, with associations being robust to air pollution adjustment.^1^, ^4^ However, another study on Danish nurses found that association of road traffic noise with dementia incidence diminished when adjusting for air pollution.^57^ Air pollution has been linked to dementia in a recent global summary report, but, critically, noise was not considered a confounder or effect modifier.^58^ This stresses that more research is needed to assess the link between noise and ADRD, and that it is necessary to consider air pollution exposure as a confounder or effect modifier, to determine whether noise effects are independent of those from air pollution (and vice versa) and whether there is a synergistic effect of these exposures on ADRD. Chronic noise exposure has been linked with slower processing speed and reduced global cognition in some cross‐sectional and longitudinal studies,^2^, ^59^ although findings on rates of cognitive decline remain mixed.^2^, ^60^, ^61^ Emerging neuroimaging research suggests that noise may contribute to lower gray matter in ADRD‐relevant regions,^62^ while biomarker evidence from blood, positron emission tomography, or autopsies are extremely limited and consequently represent a major gap in noise–ADRD knowledge.

## 3 STEP 2: SELECTION OF NOISE METRICS THAT MATCH THE PATHWAY(S) OF INTEREST

There are various ways in which noise can be measured and quantified. Noise is typically expressed as sound pressure levels in decibels (dB), a logarithmic scale in which a 10‐unit increase corresponds to a 10‐fold increase in sound intensity and is perceived as roughly twice as loud. Noise levels are often reported using A‐weighting or C‐weighting, reflecting that humans do not hear all frequencies equally. A‐weighting (dBA) approximates human hearing at low intensities, with greatest sensitivity to mid‐range frequencies and less sensitivity to low and very high frequencies. In contrast, C‐weighting (dBC) is more useful for measuring high‐intensity noise, such as loud impacts or explosions.

Noise measures can be used to create exposure metrics for epidemiologic studies, either as absolute levels or as values above or below a specified cut point (e.g., the median, 10th percentile, or 90th percentile). Perhaps the most common absolute noise metric is the A‐weighted equivalent continuous sound level over period T (LA_eq,T_). This metric represents the average noise level on the log scale, standardized over a specified period (T), such as 1, 8, or 24 hours. Another common approach is to account for the cumulative exposure to noise throughout a daywhile penalizing levels that occur in the evenings when people are at home and at night when people are commonly sleeping. For example, L_den_ applies a 10 dB penalty for noise occurring at night and a 5 dB penalty for noise occurring in the evening while the DNL (day–night level) applies only the 10 dB for nighttime hours. These penalty‐weighted metrics were developed to predict annoyance and are widely used, but the optimal noise metrics for associations with disease outcomes such as ADRD have not been established. Some researchers isolate nighttime sound levels (L_night_) as the average sound level during nighttime, defined as the period 8:00 pm to 7:00 am for DNL or 11:00 pm to 7:00 am for L_den_, which is important for the sleep disruption pathway.^13^, ^47^ The maximum sound level (L_max_) and the total energy in a sound event, normalized to a 1‐second period (SEL) are two other metrics used to quantify short‐term, high‐intensity disturbances, such as aircraft flyovers, railway horns, and occupational exposures.^15^ If high‐intensity noise disturbances are more common, noise can be characterized by the number of events exceeding a specified threshold (N_x_), such as the count of aircraft flyovers that exceed 70 dB (i.e., N_70_). Relatedly, the intermittency ratio (IR) is the proportion of sound energy from distinct events occurring above a pre‐specified threshold, commonly 3 dB above LA_eq,T_.^63^ This recently conceptualized metric may be useful for quantifying noise levels that vary in intensity over time and has some biological plausibility, though it has been used in very few health studies to date. Sound levels that are > 10% (L_10_) and 90% (L_90_) of the time are also options for metrics. L_10_ is a metric useful for quantifying intrusiveness from loud, peak noise events, whereas L_90_ is helpful for quantifying background noise levels.

When selecting noise metrics, we recommend prioritizing simple, widely available measures that align with the putative pathway(s) of interest (Table 1). LA_eq,1hr_ is critical for calculating policy‐relevant DNL and L_den_, which we stress as primary metrics for outcomes other than hearing loss and stress. For studies focused on sleep disruption, nighttime‐specific metrics (e.g., average nighttime noise, L_night_) are the most relevant and widely used, while studies examining hearing‐related pathways should incorporate measures of short‐duration peaks (e.g., L_max_) when possible. Exploratory measures that capture intermittency, frequency content, or psychoacoustic characteristics may offer additional mechanistic insight but are not currently considered essential for most epidemiologic studies.

**TABLE 1:** Primary, secondary, and exploratory noise metrics to consider for inclusion by putative pathway affecting Alzheimer's disease and related dementias outcomes.

Mechanism	Primary metric(s)	Secondary metric(s)	Exploratory metric(s)
Hearing loss	Average sound levels (e.g., LA_eq,8hr_)	Maximum sound levels (e.g., L_max_); event‐level energy metrics (e.g., SEL)	High‐frequency LA_eq,T_; frequency‐specific dose measures
Stress	Average sound levels (e.g., LA_eq,8hr_ and LA_eq,24hr_)	Nighttime average level (L_night_)	Peaks; intermittency; low‐frequency components; room façade‐specific
Mental health	DNL/L_den_ having evening/night penalties (LA_eq,1hr_ is needed to calculate)	Nighttime average level (L_night_)	Peaks; intermittency; psychoacoustic metrics (e.g., roughness, fluctuation strength); room façade‐specific
Sleep disruption	DNL/L_den_ (with evening/night penalties; LA_eq,1hr_ is needed to calculate)	Nighttime average level (L_night_)	Nighttime peak levels; nighttime intermittency ratio; speech‐like or frequency‐specific patterns; bedroom façade‐specific; number of events exceeding a threshold
Neuropathological damage	DNL/L_den_ having evening/night penalties (LA_eq,1hr_ is needed to calculate)	Nighttime average level (L_night_)	Peaks; background noise levels (e.g., L_90_); alternative frequency weighting (e.g., C); room façade‐specific

Abbreviations: DNL, day–night average sound level; L_den_, day–evening–night average sound level; L_max_, maximum sound level; L_night_, nighttime average sound level; SEL, sound exposure level.

## 4 STEP 3: IDENTIFICATION OF NOISE DATA

Selecting noise data for an ADRD study involves first understanding the main types of exposure data sources available and relevant for a given study population, whether national or strategic noise maps; detailed propagation models; custom statistical models using land‐use regression or machine learning; occupational information; or, in limited cases, personal dosimetry. Though designed for regulatory purposes, strategic maps and other large public datasets may offer sufficient exposure contrast for broad cohort studies, while finer resolution or pathway‐specific questions (e.g., sleep requiring façade‐level L_night_) may require detailed regional models and collaboration with an acoustician, noise engineer, or noise exposure scientist. Regardless of the data source, researchers should review documentation for each dataset's validation, spatial and temporal resolution, included noise sources, and available metrics to ensure alignment with the study's mechanistic pathway and exposure period. Table 2 summarizes many common data resources currently available in different places of the world.

**TABLE 2:** A sample of major noise data resources by continent.

**Dataset/source**	**Type**	**Spatial coverage**	**Spatial resolution**	**Temporal coverage**	**Temporal resolution**	**Noise metrics**	**Rapid availability**	**Notes/limitations**
**EUROPE**								
**EU Environmental Noise Directive (END) Maps** ^64^	Modeled (propagation); transportation, industry	Europe (EEA‐32 countries + UK; multi‐country)	≈ 10 m grid or building façade level (urban areas); output as noise contour maps	5‐year intervals (rounds in 2007, 2012, 2017, 2022)	Annual average (day–evening–night and night)	L_den_, L_night_ (annual averge)	Yes: Europe‐wide (EEA‐32 + UK). Publicly downloadable noise maps for 2007, 2012, 2017, 2022 rounds. Where: European Environment Agency (EEA) portal + national environment agency portals.	Comprehensive coverage of major noise sources and populations. Standard EU metrics, enabling cross‐country comparisons. Heterogeneous input data (improved by CNOSSOS‐EU standardization in latest round). Excludes smaller roads/areas below thresholds.
**United Kingdom Strategic Noise Maps** ^65^, ^66^, ^67^, ^68^	Modeled (propagation); transportation (road & rail)	England; Scotland; Wales; Northern Ireland (national)	≈ 10 m grid (4 m height receptors); available as GIS noise contours	5‐year intervals (rounds 1–4: 2007, 2012, 2017, 2022)	Annual average (long‐term day–evening–night, day, evening, night periods)	L_den_, L_night_ (annual average; also L_day_, L_evening_, LA_eq16h_)	Yes: United Kingdom. Rounds available: 2007 (major sources), 2012 (major sources), 2017 (major sources), 2022 (all sources). Where: DEFRA data portal (England), Transport Scotland, DAERA‐NI (Northern Ireland), Welsh Government.	Covers major transport sources (major roads and railways; all public roads included from Round 4). Major airports are mapped separately by operators (not included). Based on modeling (no direct measurements, per regulations). Latest round uses updated Defra model aligned with EU methods (improved accuracy, consistency). It does not include noise from so‐called minor roads, branch lines, or metro systems so will underestimate exposures.
**CNOSSOS‐EU Model** (Common Noise Assessment Methods) ^71^	Modeled (propagation); transportation, industry	Pan‐European (standard method for EU; adaptable elsewhere)	User‐defined (e.g., calculates at receptors on 10–100 m grids or building points)	Not fixed; applicable to any year (implemented from ≈ 2018 onward in EU mapping)	Annual average conditions (can model day, evening, night periods)	L_den_, L_night_, or LA_eq_ for day/evening/night periods (annual average)	No: Open methodology (published); software implementations vary (some open, some commercial). Adapted for exposure assessment in European epidemiological studies by Morley et al.^122^	Unified noise calculation framework for roads, rails, aircraft, industry. Ensures consistency and comparability across EU maps. High input data requirements (traffic, 3D geometry, etc.) and technical expertise needed. Primarily for outdoor noise mapping; does not directly address indoor exposure. Widely adopted in Europe, improving reliability of health studies linking noise exposure.
**Nordic Prediction Method (NORD2000)** ^72^	Modeled (propagation); transportation (road, rail, aircraft)	Nordic countries (Denmark, Norway, Sweden, Finland); adaptable elsewhere	Flexible; typically high resolution (building façades, 10–20 m grids) depending on input data	Not fixed; modeling method can be applied to any year (baseline reports from 2000s; in ongoing use)	Annual average; can simulate day/evening/night periods	L_den_, L_night_, LA_eq_, and other annual averages per EU/ISO standards	No: Methodology publicly documented (Nord2000 report); software implementations usually proprietary (e.g., SoundPLAN, CadnaA)	Widely used in Nordic countries before CNOSSOS‐EU adoption. Robust for complex terrain and meteorology (accounts for refraction, ground absorption, barriers). Requires high‐quality traffic and environmental inputs. Not open data by default; results depend on project sponsors.
**sonBASE Noise Database** ^73^	Modeled (propagation); transportation noise (road & rail)	Switzerland (national)	High‐resolution (modeled at building façades nationwide).	Snapshot ≈ 2015 (noise emissions of 2015); system in place for future scenarios. Historic data from 1990 also available.	Day (16 hour) and night (8 hour) annual average	L_day_, L_evening_, L_night_; convertible to L_den_, DNL (annual average)	Yes: Switzerland (national). Available open‐access for 2015 baseline (plus historic 1990). Where: opendata.swiss (WMS/GeoJSON).	Comprehensive national coverage using traffic counts on ≈ 68k km of roads and 3k km of rails. High data quality and spatial detail, enabling robust exposure assessment in research. Covers major transport noise; other sources (industry, etc.) not fully included. Lacks frequent updates (one major baseline); assumes stability over time or requires modeling changes. Only outdoor levels (most‐exposed façade).
**NORTH AMERICA**								
Canadian Urban Environmental Health Research Consortium **Noise Exposure Models** ^111^	Modeled (statistical); Transportation (road traffic focus)	Canada (national, urban emphasis)	Postal code or neighborhood level nationally (≈ 100 m scale); finer (≈ 10–30 m) in select cities	≈ 2000s–2010s snapshots (city models 2003–2015; national model ≈ 2016)	Annual average (annual day‐night)	L_den_, L_night_ (annual average)	Yes: Canada (national, urban emphasis). Models from ≈ 2003–2016 (city‐ and national‐level surfaces). Where: CANUE portal (submit request).	National noise surfaces for epidemiology, combining detailed city maps and land‐use regression modeling​. Provides standardized exposure for cohorts across Canada. Strength: fills data gap in N. America outside US. Limitations: Patchwork data (only some cities had detailed maps); relies on model extrapolation elsewhere. Primarily road noise (rail/air minor). Uncertain temporal consistency (assumes little change or uses LUR for back‐casting).
**Bureau of Transportation Statistics (BTS) National Transportation Noise Map** (US Department of Transportation) ^19^	Modeled (propagation); Transportation noise (road, aviation, rail)	National (Contiguous United States; separate data for Alaska, Hawaii)	Raster grid (≈ 30 m cells); data are mosaicked by state/region (requires mosaic repair per instructions)	2016, 2018, 2020 (updated roughly biennially; future updates envisioned annually)	Annual average; no distinct day/night split in public data	LA_eq_, 24 hour (A‐weighted equivalent continuous sound level over 24 hours); values available for individual modes and combined sources (annual average)	Yes: United States (national). Releases for 2016, 2018, 2020. Where: USDOT/BTS website.	Suitable for large‐scale exposure assessment: national coverage and consistent methodology. Can be linked to cohorts via geocoded location (e.g., census tract or coordinate) and used to estimate long‐term transport noise exposure. **Caveats**: Not precise at specific addresses (no local shielding; excludes non‐transportation noise), so use for individual‐level exposure should consider potential misclassification. Useful for comparing relative exposure or for epidemiologic studies with many US regions over time (longitudinal tracking possible), however, there are no data < 45 dB and missing data in most residential areas.
**National Park System (NPS) National Soundscape** (Geospatial Sound Model) ^69^	Modeled (statistical LUR using random forestsa); All‐source ambient noise (natural + anthropogenic)	National (contiguous US land areas)	Grid of 270 m cells covering the US landscape	≈ 2010–2015 (model published 2014; represents contemporary conditions circa early 2010s; not regularly updated; one‐time model results)	“Typical summer day” conditions; day vs. night levels were modeled separately (providing daytime and nighttime L_50_ in later analyses)	L_50_ (A‐weighted median sound level, exceeded 50% of the time) for existing ambient sound; Also available: L_10_ (90th percentile, higher noise) and L_90_ (10th percentile, background) for day/night in dataset	Yes: United States (national, continental). Represents early 2010s, published 2014. Where: NPS DataStore.	Provides a comprehensive ambient noise exposure surface including all sources (transportation, industrial, biophysical). High spatial resolution (270 m) facilitates linkage to fine geographic units (neighborhoods, census blocks). Used in health studies of noise disparities and can be aggregated to community level for epidemiology. **Limitations**: Represents a past time period (early 2010s) and uses median levels (L_50_), which may need conversion to more common metrics (e.g., DNL or LA_eq_) for some health risk estimates. Not especially representative of granular levels in urban areas and intended for natural areas. The NPS model has an empirical ceiling near 65 dB, limiting discrimination at higher exposures and potentially attenuating exposure‐response estimates in urban or high‐exposure populations.
**HowLoud “Soundscore” Map** (HowLoud, Inc., California, United States)	Modeled (proprietary algorithm; combined sources including road traffic, aircraft, and local stationary noise)	Urban areas across the United States (covering 1000+ cities and towns, including all major metro areas)	≈ 30 m (parcel level); provides a continuous noise map overlaid on street maps; can distinguish differences at the block or individual address level	≈ 2015 (initial nationwide rollout); updates not publicly documented; likely uses static inputs (traffic counts, flight paths, land use) from mid‐2010s	Day‐night average (corresponds to ≈ 24 hour average sound level); Soundscore is based on a 24‐hour average noise level with penalties for nighttime noise (similar to DNL)	Soundscore (composite index)—integrates modeled road 24‐hour LA_eq_, airport DNL, and local “hubbub” (e.g., venues, human activity) into a single 0–100 score	Yes: US urban areas (≈ 1000+ cities), but proprietary. Approx. 2015 surfaces. Where: Public web map (data licensing required for downloads).	Designed for comparing noise across neighborhoods. Has been used in real estate and urban planning contexts. It has potential for health research if data can be licensed, as the high spatial detail allows linkage to individual addresses or ≈ 100 m grids. **Limitations**: Provides an index rather than precise dB, and methodology is not fully transparent. Not commonly used in peer‐reviewed health studies yet, but could serve as a consistent nationwide noise exposure metric if obtained.
**Federal Aviation Administration Aircraft Noise Contours** (Part 150 studies) ​^112^	Modeled (propagation using FAA's Integrated Noise Model or AEDT)	Local—Airport environs for 90 of the major US airports	Varies; outputs are vector contour lines (polygons) for specified levels (not gridded); common contours: 65, 60, 55 dB (DNL)	Various years (each airport updates at ≈ 5–10 year intervals); many current maps are for the 2015–2025 period	Day‐night average sound level (DNL) over a year (24‐hour average with 10 dB night penalty)	DNL in dB—e.g., 65 dB DNL contour (often used as impact threshold for land use; annual average)	Yes: for ≈ 90 major US airports. Various 2015–2025 contour products. Where: FAA Noise Map Archive (PDFs), some GIS files available upon request.	Useful for assessing airport noise exposure for communities near airports. Health researchers have used population within 65 dB DNL contours, or distance to contour, as exposure metrics. Can link cohort residences to the nearest contour to classify exposure (e.g., ≥ 65 dB vs. lower) if geocoded accurately. High accuracy for aviation noise specifically, but limited spatial scope (only areas around airports). Not easily integrated for non‐airport areas; for national studies including aviation noise, researchers sometimes overlay multiple airport contours on population maps.
**University of Washington** Department of Environmental & Occupational Health Sciences **National Transportation Noise Exposure Map** ^113^	Modeled (propagation); transportation noise (road, rail, aviation)	National (United States)	Census tract	2023–present	Annual average (LA_eq_)	LA_eq_ > 45 dB (annual average)	Yes: DEOHS website ‐ national transportation noise weekly updates	Provides updated exposure surfaces and interactive map for transportation noise exposure. See website for details.
**Federal Highway Administration (FHWA) Traffic Noise Model (TNM)** ^114^	Modeled (propagation); road traffic noise	Any US road or highway project (user‐defined area); no pre‐generated national dataset; on‐demand modeling tool	N/A (no fixed grid; resolution depends on user‐defined receiver spacing); can produce point results at specific locations or noise contour distances from roads	N/A (user provides traffic inputs for a specific time period; no time‐series output; each run is a static scenario	Typically 1 hour	LAeq (1 hour) by default (often 1‐hour worst noise, or a 24‐hour average if traffic data provided); can compute DNL or L_den_ by combining multiple runs (day, evening, night with penalties); can compute L_10_, L_50_, L_90_ using older STAMINA model; A‐weighted dB	No: Free software (available from FHWA); no central data—users must run model with their data	While TNM is accurate for road noise, it requires detailed input data; thus it is not used to directly map large epidemiologic cohorts unless inputs are available. More common in local studies or to validate other models. For example, researchers might use TNM to calibrate/validate simpler noise estimates in a city (e.g., for a land‐use regression model). TNM underpins national road noise estimates (e.g., HowLoud, BTS map), but on its own it's a tool rather than a ready dataset
**Federal Aviation Administration Aviation Environmental Design Tool (AEDT)** ^115^	Modeled aircraft noise (propagation); computes sound propagation from aircraft operations	Any airport or airspace in United States (user defined); commonly applied at airport level or regional airspace	Flexible; outputs at specified receptor points, at dynamically derived receptor points, or as contours around airports	N/A (user provides operational scenarios for a given year or representative day); often used for annual average day or specific year forecasts	User defined	DNL contours; SEL for single events or number of events above X dB, hourly LA_eq_, and time‐above‐threshold metrics can be also obtained; A‐weighted dB	No: Free (with registration)—provided by FAA to practitioners (Windows software)	AEDT is mainly used by acoustical professionals; in health research, its outputs (e.g., contours or grid noise levels) have been used indirectly via airport studies. Not typically run by health researchers due to complexity, but its results (like population within certain DNL contours) inform health impact studies. Could be used to simulate scenarios (e.g., changes in flight operations) in research. Overall, AEDT is a tool for high‐precision airport noise exposure modeling if detailed data are available.
**Noise Job Exposure Matrix (JEM)** ^94^, ^95^	Modeled (occupational exposure matrix)	United States (national); adaptable to other countries	N/A (job‐based, not spatially mapped)	N/A (represents typical exposures by job title)	N/A	Occupational noise exposure estimates (by job title/industry); LA_eq,8h_	Yes: publicly available online; downloadable matrix	Enables assignment of occupational noise exposure in epidemiologic studies using job/industry codes. Not map‐based; complements residential/community noise data. Useful for studies considering total noise exposure (occupational + environmental). Does not provide spatial or temporal mapping; relies on accurate job coding.
**AFRICA**								
**Accra LUR Noise Model** ^22^	Modeled (statistical LUR); all‐source environmental noise	Greater Accra Metropolitan Area (GAMA), Ghana (city‐wide)	≈ 50 m grid (predictions at 4 m height); city‐wide surface	2019–2020 (hybrid monitoring campaign; 136 sites measured for 7 days; 10 sites measured for 1 year)	Annual averages (hourly, daily, day–evening–night, and night)	LA_eq,24h_, L_den_, L_night_, Intermittency Ratio (IR) (annual average)	Yes: Exposure surfaces available. Where: https://zenodo.org/records/11223249. from Imperial College London/Pathways to Equitable Healthy Cities consortium.	First comprehensive city‐wide noise model in SSA. High predictive accuracy. Captures all‐source environmental noise, not just transportation. Limitations: single‐city coverage; LUR may not transfer to other SSA cities without local calibration. No source separation.
**South Africa Informal Settlements LUR** ^116^	Modeled (statistical LUR); all‐source environmental noise	Western Cape Province, South Africa (4 informal settlement areas)	Site‐level predictions (≈ 100–200 m based on measurement density)	Summer 2015–2016 (week‐long rotating measurements)	Annual average (estimated from summer campaign)	L_den_ (annual average)	No: Research model developed for a specific cohort study (air pollution/respiratory health). Data availability by request from authors.	First published noise LUR in SSA. Developed alongside an air pollution health study, enabling co‐exposure analysis. Limitations: limited to 4 informal settlement areas; summer‐only measurements may not capture seasonal variation; model not validated externally.
**Kampala and Entebbe Noise Dataset** ^117^	Measured (mobile‐based SLM + audio); all‐source ambient noise	Kampala (5 divisions) and Entebbe (4 wards), Uganda	Point samples with GPS coordinates; suitable for spatial aggregation	2024–2025 (continuous collection)	Momentary measurements (short audio recordings with SPL)	LA_eq_ (short‐term, per sample)	Yes: Open‐access dataset. Where: Scientific Data (Nature).	Largest existing urban sound dataset from an African city (61,821 annotated samples). Includes geolocated audio recordings enabling ML‐based source classification. Useful for model training and validation. Limitations: not a predictive exposure model; coverage depends on sampling routes; not designed for epidemiologic exposure assignment without further modeling.
**ASIA**								
**India CPCB National Ambient Noise Monitoring Network (NANMN)** ^118^	Measured (continuous SLM); all‐source ambient noise	India (national, 7 metro cities: Delhi, Mumbai, Kolkata, Chennai, Bangalore, Lucknow, Hyderabad); also 345 manual monitoring locations in 129 cities	Fixed‐point monitors (70 continuous stations; 10 per metro city, categorized by zone)	2011–present (continuous, 24/7)	Continuous (real‐time); annual summary reports	LA_eq_ (continuous); day and night zone‐specific standards	Yes: India (national). Where: CPCB portal (cpcb.nic.in); annual reports publicly available.	Only national‐scale continuous noise monitoring network in South Asia. Covers 7 major metros with zone‐classified stations (industrial, commercial, residential, silence). Useful for temporal trend analysis and zone‐level exposure classification. Limitations: point monitors, not a spatial model; cannot assign exposure at residential address level; limited to major cities; manual network covers more cities but with intermittent sampling.
**Shanghai LUR Noise Model** ^25^	Modeled (statistical LUR); all‐source environmental noise	Shanghai, China (city‐wide)	City‐wide surface (resolution depends on predictor grid; measurements at 144 sites)	2019 (3 seasons: winter, spring, summer)	Period‐specific (morning, afternoon, evening per season)	LA_eq,40min_ (by period)	No: Research model. Where: available from study investigators upon request.	Multi‐season, multi‐period LUR for a major Chinese megacity. Integrates land‐use, road networks, and socioeconomic variables. Good predictive accuracy via 10‐fold cross‐validation. Limitations: single‐city; no nighttime measurements reported; 40‐minute measurement duration may not capture full temporal variability; no L_night_ or L_den_ directly.
**Taichung LUR Noise Model** ^24^	Modeled (statistical LUR); road traffic noise	Taichung, Taiwan (metropolitan area)	City‐wide surface (50 monitoring stations; high‐resolution predictor data)	2013–2014 (yearlong continuous monitoring)	Annual averages (24 hour and nighttime)	LA_eq,24h_, L_night_; octave‐band frequency components (31.5–8000 Hz)	No: Research model. Where: available from study investigators.	Unique frequency‐specific LUR enabling pathway‐specific analyses (e.g., hearing loss at 4000 Hz vs. cardiovascular at 125 Hz). Yearlong continuous data at 50 sites. Good validation (RMSE 2.09 dBA for 24 hours). Limitations: single city; road traffic focus (does not separate other sources); based on 2013–2014 data.
**Guangzhou LUR Noise Model** ^26^	Modeled (statistical LUR); all‐source environmental noise	Guangzhou, China (municipality‐wide)	City‐wide surface (100 monitoring sites, population‐density stratified)	Two measurement campaigns, reported as winter/spring and summer/autumn (study period given as Dec 2022–May 2023 in source)	Daytime (morning, afternoon, evening) and nighttime	LA_eq_ (daytime periods and nighttime separately)	No: Research model. Where: available from study investigators.	Separate daytime and nighttime noise maps for one of Asia's most densely populated cities. Population‐density stratified site selection. Limitations: single city; ≈ 6‐month campaign (not full year); 30‐minute measurements per site per period; no L_den_ or annual average computed directly.
**Dalian LUR Noise Model** ^119^	Modeled (statistical LUR); all‐source environmental noise	Dalian, China (municipality)	City‐wide surface (202 measurement sites: 172 routine + 30 purpose‐designed; 200 m grid)	≈ 2009–2010 (monitoring campaign)	Daytime averages	LA_eq_ (daytime)	No: Research model (historical).	First‐ever application of LUR methodology to noise modeling. Explained 83.2% of spatial variability. Foundational for subsequent noise LUR studies globally. Limitations: historical (early 2010s); daytime only; no nighttime or 24 hour metrics; single city; superseded by more recent Chinese LUR models.
**OCEANIA**								
**Ambient Maps Transport Noise (AURIN)** ^74^	Modeled (propagation); Transportation noise (road + rail). Uses SoundPLAN	Australia (nationwide, multi‐city)	Building‐level (façade noise at every building and floor); also ≈ 10 m raster overlay	2023 (current release; future updates TBD)	Day, evening, night averages (and 24 hour composite)	L_day_, L_evening_, L_night_ (and combined metrics like L_den_)	Yes: Australia (major cities + nationwide building‐level surfaces). Release year: 2023 dataset available. Where: AURIN (restricted academic access).	First continent‐scale noise map with high detail. Provides exposure by time of day and source for most populated areas. Valuable for national health studies; however, data access requires permission/payment. Focuses on transport sources (less on ambient community noise). No historical data yet for trends. Outdoor levels at façades.
**INTERNATIONAL**								
**NoiseCapture App Data (Noise‐Planet)** ^120^	Measured; All‐source ambient (crowdsourced)	International (user‐contributed locations; variable coverage)	Point samples (smartphone GPS locations; aggregated to street‐level heatmaps)	2016–present (continuous collection; real time)	Momentary measurements (seconds to minutes; can aggregate to pseudo‐averages)	LA_eq_ (short term; user recordings)	Yes: Open access (online maps; data via API)	Community‐driven noise measurements mapped worldwide. Captures actual sound levels including all local sources. Useful for awareness, hotspot identification, and model validation. However, data are unstructured—spatial coverage is sparse and biased to where volunteers go. Lacks standardized time averaging (not an official L_den_ or L_night_). Not particularly suitable as sole exposure measure in epidemiology due to representativeness issues.
**NoiseModelling Platform** ^121^	Modeled (propagation); Open‐source GIS tool (uses CNOSSOS‐EU)	International (user‐driven; used in EU, NZ, etc.)	User‐dependent (can produce fine‐scale maps for entire cities; receiver grids at few‐meter spacing)	N/A (tool, not a fixed dataset; available since ≈ 2018)	User‐defined (typically computes annual L_den,_ L_night_)	L_den_, L_night_, LA_eq_ (flexible per scenario)	No: Open‐source software (MIT License; free to use)	Free tool for generating custom noise maps. Enables large‐scale urban noise simulations without proprietary software. Useful for research and teaching; has been applied to city noise projects. Data inputs from open sources (OSM, etc.) can be used, but output quality hinges on input accuracy. Not a ready‐made dataset—requires significant data collection and computing. Facilitates experimentation but less commonly used for big epidemiologic cohorts (due to effort required per study).
**Computer Aided Noise Abatement (CadnaA) Software** (DataKustik GmbH, Greifenberg, Germany)	Modeling software (propagation); commercial noise mapping tool	International (projects in many countries; user‐defined scope)	User defined (from individual sites to nationwide maps; supports high‐resolution grids and 3D geometry)	N/A (software, in use since late 1980s; updated regularly)	User defined (can model specific hours, day/evening/night, or annual averages)	Any (supports L_den_, L_night_, LA_eq_, L_max_, etc., per supported standards)	No: Proprietary (commercial license required from DataKustik GmbH, Greifenberg, Germany)	Widely used commercial platform for environmental noise prediction. Supports multiple standards (e.g., CNOSSOS‐EU, NORD2000, ISO 9613) and detailed modeling (terrain, reflections, barriers). Has powered many official noise maps and studies worldwide. Limitations: high cost and expertise needed, so mainly used by government agencies and consultants; output data not public unless released by clients. Not directly accessible to researchers without collaboration.
**SoundPLAN Software** (SoundPLAN GmbH, Backnang, Germany)	Modeling software (propagation); commercial noise mapping tool	International (projects in many countries; user‐defined scope)	User defined (from individual sites to nationwide maps; supports high‐resolution grids, façade receptors, and 3D geometry)	N/A (software, in use since mid‐1980s; updated regularly)	User defined (can model specific hours, day/evening/night, or annual averages)	Any (supports L_den_, L_night_, LA_eq_, L_max_, etc., per supported standards)	No: Proprietary (commercial license required from SoundPLAN GmbH, Backnang, Germany)	Widely used commercial platform for environmental noise prediction. Supports multiple standards (e.g., CNOSSOS‐EU, NORD2000, ISO 9613) and detailed modeling (terrain, reflections, barriers). Powers large national and regional mapping efforts (e.g., AURIN Ambient Maps in Australia). Limitations: high cost and expertise needed, so mainly used by government agencies and consultants; output data not public unless released by clients. Not directly accessible to researchers without collaboration.

*Note*: There are many city‐specific statistical noise models that may be available locally but are excluded here for simplicity.

Abbreviations: CNOSSOS‐EU, European Union Common Noise Assessment Methods; DNL, day–night average sound level; GIS, geographic information system; ISO, International Standards Organization; L_den_, day–evening–night average sound level; L_max_, maximum sound level; L_night_, nighttime sound average level; LUR, land use regression; ML, machine learning; SEL, sound exposure level; SLM, sound level meter; SPL, sound pressure level; SSA, sub‐Saharan Africa.

There are many existing strategic noise maps and national models already available in several countries. These resources include the EU END maps,^64^ UK strategic noise maps,^65^, ^66^, ^67^, ^68^ the US National Transportation Noise Map,^19^ and the US National Park Service National Soundscape Model.^69^ The strengths of such existing, large‐scale geospatial data resources are their comprehensive spatial coverage, standardized methods, and public availability. However, they can be limited by their coarse resolution, exclusion of certain features (e.g., minor roads), frequent lack of validation evidence, reliance on older base years,^6^, ^18^, ^70^ and lack of consideration of non‐spatial factors (e.g., personal behaviors and activities). Additionally, these maps typically report noise exposure only above specific thresholds, such as 55 dB in the EU END, which can be problematic, because large epidemiological studies have found that health effects and exposure–response relationships begin at lower noise levels, often starting from 35 to 40 dB.^13^

In some places, there are city or regional propagation (alternatively: physical, deterministic, source–path–receiver) models available that are currently the gold standard of noise models due to their highly accurate estimation of sound source–path–receiver relationships, noise source specificity (e.g., road, aircraft), and their high resolution (e.g., façade level). Yet, these are not yet widely applied outside certain locations due to their rich and harmonized input data required. Examples of propagation models include the EU Common Noise Assessment Methods (CNOSSOS‐EU),^71^ Nord2000,^72^ Swiss Noise Database (sonBASE),^73^ and Australian Urban Research Infrastructure Network (AURIN).^74^

When propagation models are infeasible, noise exposure scientists often develop location‐specific statistical models (alternatively: empirical, stochastic) in land use regression^25^, ^75^, ^76^ or machine learning–based frameworks.^77^, ^78^, ^79^ Statistical models are flexible and scalable. However, they require noise measurements for model training and validation, cannot distinguish between different sources (e.g., community noise vs. road noise in the community), and have traditionally been considered less accurate due to their empirical nature—which has led to limited development of some hybrid propagation–statistical approaches to leverage benefits from both approaches.^80^, ^81^ Yet, incorporating modern, high‐resolution data streams and artificial intelligence processing may enhance their accuracy, validity, and source specificity in the future.

Personal dosimetry is predominantly used in occupational studies or those with smaller relative sample sizes. It centers on direct, individual‐level measurement using wearable sound level meters^82^ and represents the gold standard for noise exposure estimation, as it considers non‐spatial factors such as personal behaviors and can incorporate both community and occupational exposures. But there are limitations associated with dosimetry, including high cost, unique sources of error, burdensome devices, and a historical lack of scalability to large cohorts and long durations. However, smartphone‐based sound level meter applications are beginning to address this scalability gap, with the National Institute for Occupational Safety and Health Sound Level Meter app demonstrating accuracy within ± 2 dBA of reference instruments using built‐in microphones and ± 1 dBA with calibrated external microphones.^83^, ^84^, ^85^ Consumer wearable devices such as smartwatches can also passively log environmental sound levels throughout the day.^86^ While these tools offer potential to scale personal noise monitoring to large cohorts at low cost, variability across devices, lack of compliance with international standards (IEC 61672), the absence of standardized calibration protocols, shorter measurement periods, and additional sources of error currently limit their use in epidemiologic research.^83^, ^86^

### 4.1 When is it okay to use public maps?

Existing strategic noise maps are generally suitable when noise is not the primary exposure or when large national cohorts require consistent coverage. They are appropriate if publicly available metrics align with your study's mechanistic pathways (e.g., L_den_ for policy‐relevant analyses), and when the maps are recent enough to match the cohort baseline.^87^ However, spatially imprecise maps may misclassify, and therefore bias, exposure estimates.^88^

### 4.2 When more advanced approaches are necessary, what should you do?

Noise exposure scientists and acousticians can help to develop a plan to generate and assign exposures from existing or new propagation, statistical, or hybrid models.^71^, ^80^, ^89^ However, it is important to reiterate that statistical modeling approaches require measured data for training and validation unless there is an existing model that is generalizable to the study area, which is usually unlikely. A noise expert may also help to integrate population mobility or dynamic exposures.^90^ Generally, when interpreting datasets whose assumptions, geometry, or local propagation components are complex, a noise expert should be integrated.^6^

### 4.3 What do you look for in map or model documentation?

It is important to identify and assess model validation results from field comparisons via metrics such as percent variability explained, correlation, and bias. Model year and temporal relevance is additionally important to seek out, as noise exposures need to align with baseline and follow‐up (many maps use static years).^87^, ^91^ A researcher should further identify the spatial resolution of a noise resource—whether grid size or, ideally, façade‐level estimates are available^88^—and consider the temporal stability of noise estimates across space.^91^ If it is critical to the research question to identify the source of noise relevant to the ADRD research question (e.g., road traffic noise), applicable sources should be investigated in the noise map or model documentation.^70^ Any assumptions made by an underlying noise model (e.g., propagation affected by meteorology, ground effects, reflections, etc.) should be investigated and their impacts on noise estimates determined.^71^, ^72^

## 5 STEP 4: ASSIGNMENT OF NOISE EXPOSURES TO PARTICIPANTS

Once noise data are obtained, exposure estimates need to be assigned to participants in an ADRD study. The main considerations when assigning noise exposures to participants are spatial assignment, temporal assignment, and participant mobility and residential histories.

Spatial noise exposure assignment is commonly done at the home address level in cohort studies for which residential address information is available. This is often accomplished by spatially overlaying noise data (e.g., raster grids, polygon noise contours) onto participant residential address points and assigning the corresponding noise estimate to each location. When granular noise data are available, more precise estimates within a single residential address can be assigned, sometimes even to each façade (e.g., most‐exposed side nearest a road, quiet side opposite a road) and floor, such as targeting a participant's bedroom or living room. Façade‐level exposure assignment reduces misclassification bias, particularly for sleep‐related pathways, because noise at the quiet façade of a building can be 10 to 20 dB lower than at the most‐exposed façade.^88^, ^92^

Researchers also need to identify appropriate temporal windows for assigning noise exposures. The main temporal dimensions that should be considered are the study years and the etiologically relevant windows. The first temporal aspect is straightforward: the primary goal is to align the noise model year with the cohort years, although noise estimates have been shown to be fairly stable over time^91^ in the absence of major changes in noise sources. Temporally aligning noise estimates to a study is not necessarily easy in practice, especially because long‐term routine noise monitoring is often unavailable to calibrate and evaluate models, unlike the situation for air pollutants in some countries. This may involve gathering historical data and/or developing approaches to reconstruct historical noise estimates^87^ if needed. The second temporal dimension—matching noise data to etiologically relevant windows—is less clear due to the lack of consensus on appropriate exposure periods. Ideally, ADRD researchers would adopt a life course perspective that would include exposure periods many decades prior to ADRD diagnoses in incidence studies,^33^, ^93^ but that may not be realistic with data constraints. Intermediate ADRD‐relevant phenotypes may emerge earlier in life through specific pathways (e.g., lifestyle and behaviors) and may have age‐specific windows of prominent influence.^32^, ^36^, ^47^ When considering long‐term, cumulative noise exposures, it is important to consider whether cumulative averages or other metrics best represent noise exposure relative to the putative pathway(s).^14^, ^34^, ^35^, ^54^ It is also recommended to conduct sensitivity analyses on different noise exposure lag periods to help clarify etiologically relevant exposures over time.

A final major factor to consider in noise exposure assignment is how to account for participant mobility, including occupational and residential exposures over time.^15^, ^94^, ^95^ Noise exposure misclassification increases if participants’ actual time spent in high versus low noise areas systematically differs from overly simplistic assumptions about their mobility (e.g., assuming a person always spends their entire day at their residence).^96^ As such, full residential histories are preferable to single addresses at study baseline, especially for diseases with long latency periods like ADRD. Considering occupational noise exposure is especially important for the hearing‐loss pathway and potentially others.^97^, ^98^ Time–activity patterns usually include time spent commuting or traveling via various transportation modes to school, recreation, or other places, and individuals can be exposed to noise during these periods as well.^90^

## 6 STEP 5: STUDY DESIGN GUARDRAILS AND ANALYTIC CONSIDERATIONS

ADRD has a long latency and multiple potential pathways, so even small design biases over time can seriously distort results. We therefore propose guardrails and analytic considerations, including: nighttime noise and granular temporal patterns, treating outdoor noise as a proxy for true exposure, integrating co‐exposures, avoiding spatiotemporal misalignment and “transference of effect,” accurately aggregating decibel units, investigating effect modifiers, and identifying selection biases.

Nighttime noise is particularly relevant for ADRD pathways because sleep disruption is one of the key mechanisms linking noise to neurobiological changes. Evidence shows that L_night_ is strongly associated with sleep disturbance and stress hormone dysregulation, and that intermittent nighttime events—captured by L_max_, N_x_, IR_night_, and similar metrics—can trigger microarousals even when average levels are modest.^34^, ^35^, ^47^, ^63^ The primary metrics used for policy decisions, DNL and L_den_, incorporate penalties for evening and nighttime noise, but the weighting is determined by levels of relative annoyance and offers no within‐evening or within‐night specificity (each evening and night hour is treated the same, respectively). The relationship between objective noise levels and subjective annoyance has been extensively characterized by established exposure–response functions, which show the proportion of a population highly annoyed rising non‐linearly with L_den_ and varying markedly by source (aircraft > rail > road at equivalent levels).^13^, ^99^ For ADRD, this matters on two counts: annoyance may itself mediate stress and sleep pathways, and L_den_ was calibrated to annoyance rather than health outcomes.^100^ Because bedrooms often face quieter façades, relying on a single façade‐level estimate may misclassify nighttime exposure, making room‐ or façade‐specific assignment important in sleep‐focused research.^88^, ^92^ When feasible for ADRD studies, researchers should therefore include at least one nighttime‐specific metric and, as secondary analyses incorporate event‐based indicators to capture temporal patterns that average metrics cannot.

Because individuals spend most of their time indoors, it is essential to recognize that outdoor noise may imperfectly represent actual dose.^96^, ^101^ Noise experienced indoors from outdoor sources varies substantially depending on building insulation, window position, façade orientation, and room location, meaning that reliance solely on outdoor estimates may introduce systematic misclassification, particularly for sleep‐related pathways.^88^, ^92^ When estimating indoor levels is infeasible, using façade‐ and floor‐specific modeled values provides a more biologically relevant approximation of nighttime exposure. Another option is for researchers to collect some basic housing or behavioral information to better capture indoor exposure variability or to evaluate effect modification. For example, bedroom orientation and floor can be a simple method to capture some of this nuance. In addition to outdoor sources, indoor noise from household appliances, electronics, conversations, building systems, and neighbors can contribute substantially to overall exposure^16^, ^17^ and may be particularly relevant for certain ADRD pathways. Future studies may want to consider both indoor and outdoor sources when assessing total noise exposure.

It is also critical to remember that energy‐ or pressure‐based noise metrics are measured on the log scale. Such metrics, measured in dB, must therefore be “un‐logged” prior to aggregation (e.g., spatial or temporal). It is invalid to average dB values over space or time; instead, the un‐logged pressure or energy values must be aggregated and then re‐logged for validity. Moreover, sound attenuates non‐linearly with distance,^102^ and averaging across heterogeneous areas can misrepresent individual exposure. Address‐level estimates are preferred; when only aggregated values are available, the spatial unit should be reported and tested in sensitivity analyses. Models with an exposure floor (e.g., the US Department of Transportation National Transportation Noise Map at ≈ 45 dB)^19^ return zeros for unmodeled areas. Excluding these observations risks selection bias, because residents of quieter areas often differ systematically from those near modeled sources. We recommend retaining low or zero values in primary analyses—as a distinct category, via distributional imputation, or estimated from a disjointed exposure–response model—with sensitivity analyses conducted accordingly.

As noise often co‐occurs with other environmental and social stressors, analyses should examine its independent effects as well as potential synergistic or interacting influences. Noise is a primary exposure and independent risk factor for ADRD, but it is likely an oversimplification to consider it in isolation. Transportation noise, in particular, co‐occurs with traffic‐related air pollution because sources of each are shared, and both may act on similar mechanisms (e.g., oxidative stress, inflammation).^1^, ^52^, ^54^, ^57^, ^103^, ^104^ Therefore, care in the design or analytic phase of a project is required to isolate the independent impacts of noise. It may also be important to investigate any potential mechanistic or biological synergisms on different scales (e.g., additive, multiplicative). Once again, exposome or life‐course frameworks could guide such comprehensive analyses.^93^

If noise exposures, potential confounders, covariates, and ADRD outcomes are spatiotemporally misaligned in the sense that they are measured on different scales, a true signal between exposure and outcome can be lost or result in a biased estimate.^105^, ^106^ To accurately partition the effect of noise from other socio‐environmental factors (e.g., traffic‐related air pollution, a major confounder) on ADRD outcomes, harmonization of exposure and confounder scale is critical to prevent “transference of effect” or “attribution shift” from one factor to another.^107^ Minimizing spatiotemporal imprecision of all variables (noise exposures, confounders, covariates, ADRD outcomes) is another important objective.^105^

Finally, it is important to consider potential effect modifiers and selection mechanisms. Factors such as sex, noise sensitivity, hearing status, air pollution, mobility, socioeconomic status, major chronic disease comorbidities, and built environment features may modify individual‐level associations between noise and ADRD outcomes.^1^, ^5^, ^108^, ^109^

## 7 STEP 6: REPORTING AND INTERPRETATION

Noise exposure research on ADRD demands careful attention to analytic and reporting practices that ensure results are interpretable, comparable, and biologically meaningful. As the field grows, a consistent set of recommendations can help researchers navigate decisions about exposure relevance, model selection, and reporting transparency. These guideposts and guardrails aim to strengthen methodological rigor while supporting harmonization across studies and facilitating translation of findings into policy and practice.

Noise should be approached as a biologically active exposure capable of influencing multiple ADRD‐related pathways, rather than as merely a nuisance or secondary environmental factor. It is becoming clearer that environmental noise is a modifiable risk factor for ADRD and that the hearing loss mechanistic pathway is only one of many likely causal pathways.^14^, ^38^ It is, quite simply, more than an annoyance and should be treated as such.

Accurate noise exposure assessment relies on using well‐validated propagation or statistical models—or personal dosimetry when appropriate—to ensure credible estimates for ADRD epidemiology. There are many validated geospatial noise models in existence, particularly in Europe where there are many accurate models (open‐source and commercial) of noise sources, paths or propagation, and receivers.^19^, ^64^, ^71^, ^72^, ^73^ Personal dosimetry, particularly in occupational settings or when there are highly variable local sources, is desirable for accurate individual level exposure classification, though this is less commonly used due to practical constraints.^15^ Partnering with exposure scientists, acousticians, engineers, and/or environmental noise exposure scientists is highly recommended to facilitate access to appropriate data and resources, model interpretation, and error estimation.

Clear and comprehensive reporting of noise exposure metrics, data sources, modeling assumptions, and assignment methods is essential for reproducibility and for enabling meaningful cross‐study comparisons. For transparency, it is critical to clearly articulate the details about noise exposure metrics used in ADRD research. The noise source or setting (e.g., total, road, aircraft, transportation, occupational), metric (e.g., LA_eq_,_T_, DNL, L_night_), time window, spatial assignment method (e.g., residential address, quiet‐side façade), and any exposure harmonization efforts over time, space, or data sources should be provided in detail. It is also critically important to clearly acknowledge the assumptions, constraints, and validation of proprietary or publicly accessible exposure models.

Researchers should also be attentive to privacy and data security considerations when working with noise exposure data. High‐resolution noise estimates linked to residential addresses, particularly at the façade or floor level, can increase the risk of participant re‐identification when combined with other geospatial datasets. Global Positioning System–tracked personal dosimetry data present additional concerns, as continuous location traces can reveal sensitive information about daily routines and movements. Noise exposure data should therefore be managed in accordance with institutional data governance requirements, including appropriate de‐identification, secure storage, and controlled access protocols, consistent with standards for other georeferenced environmental health data.

## 8 CONCLUSION

Noise is an important, modifiable, and biologically supported environmental risk factor for ADRD, with growing evidence and interest in its etiologic role. This primer offers ADRD researchers a stepwise framework for integrating noise into epidemiologic studies—from clarifying mechanistic pathways and selecting metrics, to obtaining and assigning noise data, and minimizing exposure misclassification. While L_den_ and DNL remain primary exposure metrics, attention should also be given to nighttime noise, intermittent noise events, indoor–outdoor differences, occupational, and life course exposures to capture etiologically meaningful patterns. As the field advances, improved data resources, validation practices, and integration of co‐exposures and biomarkers will strengthen causal inference and support comprehensive ADRD prevention strategies. Recognizing noise as more than a nuisance is essential for understanding and mitigating its contribution to unexplained ADRD risk.

## CONFLICT OF INTEREST STATEMENT

The authors declare no conflicts of interest. Author disclosures are available in the supporting information.

## Supporting information




**Supporting Information**: alz71513‐sup‐0001‐tableS1.docx

**Supporting Information**: alz71513‐sup‐0002‐SuppMat.pdf
