# Supplementary material for: Integrating noise as a risk factor in studies of Alzheimer's disease and dementia: Guidance for epidemiologic research
Source: Alzheimers Dement. 2026 May 22;22(5):e71513. doi: 10.1002/alz.71513 (PMC13239928; doi:10.1002/alz.71513)
Supplement: Supplementary file 1 — Supporting Information: alz71513‐sup‐0001‐tableS1.docx [file ALZ-22-e71513-s002.docx]

**Supplemental Table 1. Summary of in vivo animal studies supporting a causal role of chronic noise exposure in cognitive impairment and Alzheimer's disease-like neuropathology.**

| **Author, Year** | **Animal Model** | **Noise Protocol (type, intensity, duration)** | **Cognitive / Neuropathological Outcome** | **Proposed Mechanism** |
| --- | --- | --- | --- | --- |
| Cui et al., 2012 [1] | Wistar rats (male, adult) | White noise, 100 dB SPL, 4 h/day, 30 consecutive days | Persistent tau hyperphosphorylation (Ser199/202, Thr205, Ser396, Ser404) and NFT-like tau formation in hippocampus and prefrontal cortex; effects persisted 14+ days post-exposure. | GSK3β and phosphatase dysregulation; NMDA receptor-mediated excitotoxicity |
| Cui et al., 2015 [2] | Sprague-Dawley rats (male, adult) | White noise, 95 dB SPL, 4 h/day, 30 consecutive days | Progressive overproduction of Aβ in hippocampus persisting 7-14 days post-exposure; upregulated APP, β- and γ-secretase expression; increased TNF-α and RAGE; glial activation (GFAP, Iba-1). | Cumulative Aβ pathology via APP cleavage dysregulation; neuroinflammation (astrocyte and microglial activation) |
| Manikandan et al., 2006 [3] | Wistar rats (male, adult) | White noise, 100 dB SPL, 4 h/day, 30 days | Impaired spatial memory (radial arm maze); dendritic atrophy and reduced spine density in hippocampal CA1 and medial prefrontal cortex neurons. | Free radical imbalance (elevated MDA, decreased SOD and catalase); oxidative stress-driven neuronal structural damage |
| Cheng et al., 2011 [4] | ICR mice (male, adult) | White noise, 80 dB SPL, 2 h/day, 1–3 months | Impaired learning and memory (Morris water maze); effects at moderate (non-damaging) noise levels. | NMDA receptor subunit (NR2B) downregulation; oxidative stress in hippocampus |
| Gai et al., 2017) [5] | Wistar rats (male, adult) | White noise, 95 dB SPL, 4 h/day, 30 consecutive days | Elevated CRF and CRFR1 expression in hippocampus; delayed CRFR2 upregulation; tau hyperphosphorylation at Ser396 and Thr205 co-localized with CRF. | HPA axis / corticotropin-releasing factor (CRF) system dysregulation mediating tau phosphorylation |
| Münzel et al., 2017 [6] | C57BL/6J mice (male, adult) | Recorded aircraft noise, mean ~72 dB(A), intermittent events, exposure during sleep phase, 1–4 days | Vascular endothelial dysfunction; increased oxidative stress markers (superoxide); cerebral inflammation. Not a chronic ADRD model but demonstrates acute neurovascular effects. | eNOS uncoupling; NADPH oxidase activation; vascular oxidative stress and inflammation |
| Su et al., 2018 [7] | SAMP8 mice (senescence-accelerated, male, 3-month-old) | White noise, 85 or 98 dB SPL, 4 h/day, 30 consecutive days | Aβ overproduction and tau hyperphosphorylation (Ser396, Thr205, Thr231) in hippocampus and PFC; effects in young SAMP8 comparable to aged controls; dose-dependent with noise intensity. | Wnt/β-catenin signaling pathway inhibition; environment–gene interaction accelerating senescence |
| Cui et al., 2018 [7] | SAMP8 mice (senescence-accelerated, male) | White noise, 98 dB SPL, 4 h/day, 30 consecutive days | Hippocampal Aβ accumulation; cognitive impairment (Morris water maze); gut microbiota dysbiosis; increased intestinal and blood-brain barrier permeability. | Microbiome–gut–brain axis disruption; systemic inflammation propagating to CNS |
| Paciello et al., 2021 [8] | 3×Tg-AD mice and wild-type controls (C57BL/6, male/female) | Noise exposure causing hearing loss (protocol details in original; broadband noise, high intensity) | Exacerbated tau hyperphosphorylation (pTauSer396) in hippocampus of AD mice; increased TNF-α, IL-1β, and oxidative stress (4-HNE); accelerated cognitive decline vs. non-exposed AD mice. | Auditory sensory deprivation compounding AD pathology; impaired endogenous antioxidant (HO-1) response in AD model |
| Jafari et al., 2019 [9] | 3×Tg-AD mice and wild-type controls (male/female) | Prenatal noise stress (gestational days 15–17; intermittent noise, variable intensity) | Accelerated Aβ plaque deposition, earlier onset of cognitive decline in prenatally stressed AD mice vs. controls. | Prenatal HPA axis programming; developmental vulnerability window for AD pathology |
| Liu et al., 2016 [10] | C57BL/6 mice (male, adult) | Broadband noise, 123 dB SPL, 2 h (acute exposure inducing permanent hearing loss) | Impaired spatial learning and memory (Morris water maze); reduced hippocampal neurogenesis (BrdU+ and DCX+ cells in dentate gyrus). | Noise-induced hearing loss led to reduced hippocampal neurogenesis and synaptic plasticity |

**Supplemental References**

[1] Cui B, Zhu L, She X, Wu M, Ma Q, Wang T, et al. Chronic noise exposure causes persistence of tau hyperphosphorylation and formation of NFT tau in the rat hippocampus and prefrontal cortex. Exp Neurol 2012;238:122–9. https://doi.org/10.1016/j.expneurol.2012.08.028.

[2] Cui B, Li K, Gai Z, She X, Zhang N, Xu C, et al. Chronic Noise Exposure Acts Cumulatively to Exacerbate Alzheimer’s Disease-Like Amyloid-β Pathology and Neuroinflammation in the Rat Hippocampus. Sci Rep 2015;5:12943. https://doi.org/10.1038/srep12943.

[3] Manikandan S, Padma MK, Srikumar R, Jeya Parthasarathy N, Muthuvel A, Devi RS. Effects of chronic noise stress on spatial memory of rats in relation to neuronal dendritic alteration and free radical-imbalance in hippocampus and medial prefrontal cortex. Neurosci Lett 2006;399:17–22. https://doi.org/10.1016/j.neulet.2006.01.037.

[4] Cheng L, Wang S-H, Chen Q-C, Liao X-M. Moderate noise induced cognition impairment of mice and its underlying mechanisms. Physiol Behav 2011;104:981–8. https://doi.org/10.1016/j.physbeh.2011.06.018.

[5] Gai Z, Su D, Wang Y, Li W, Cui B, Li K, et al. Effects of chronic noise on the corticotropin-releasing factor system in the rat hippocampus: relevance to Alzheimer’s disease-like tau hyperphosphorylation. Environ Health Prev Med 2017;22:79. https://doi.org/10.1186/s12199-017-0686-8.

[6] Münzel T, Daiber A, Steven S, Tran LP, Ullmann E, Kossmann S, et al. Effects of noise on vascular function, oxidative stress, and inflammation: mechanistic insight from studies in mice. Eur Heart J 2017;38:2838–49. https://doi.org/10.1093/eurheartj/ehx081.

[7] Su D, Li W, She X, Chen X, Zhai Q, Cui B, et al. Chronic noise exposure exacerbates AD-like neuropathology in SAMP8 mice in relation to Wnt signaling in the PFC and hippocampus. Sci Rep 2018;8:14622. https://doi.org/10.1038/s41598-018-32948-4.

[8] Paciello F, Rinaudo M, Longo V, Cocco S, Conforto G, Pisani A, et al. Auditory sensory deprivation induced by noise exposure exacerbates cognitive decline in a mouse model of Alzheimer’s disease. eLife 2021;10:e70908. https://doi.org/10.7554/eLife.70908.

[9] Jafari Z, Okuma M, Karem H, Mehla J, Kolb BE, Mohajerani MH. Prenatal noise stress aggravates cognitive decline and the onset and progression of beta amyloid pathology in a mouse model of Alzheimer’s disease. Neurobiol Aging 2019;77:66–86. https://doi.org/10.1016/j.neurobiolaging.2019.01.019.

[10] Liu L, Shen P, He T, Chang Y, Shi L, Tao S, et al. Noise induced hearing loss impairs spatial learning/memory and hippocampal neurogenesis in mice. Sci Rep 2016;6:20374. https://doi.org/10.1038/srep20374.
